# Multivalent exposure of trastuzumab on iron oxide nanoparticles improves antitumor potential and reduces resistance in HER2-positive breast cancer cells

**DOI:** 10.1038/s41598-018-24968-x

**Published:** 2018-04-26

**Authors:** Marta Truffi, Miriam Colombo, Luca Sorrentino, Laura Pandolfi, Serena Mazzucchelli, Francesco Pappalardo, Chiara Pacini, Raffaele Allevi, Arianna Bonizzi, Fabio Corsi, Davide Prosperi

**Affiliations:** 1 Department of Biomedical and Clinical Sciences “L. Sacco”, University of Milano, via G. B. Grassi 74, 20157 Milano, Italy; 2 NanoBioLab, Department of Biotechnologies and Biosciences, University of Milano-Bicocca, Piazza della Scienza 2, 20126 Milano, Italy; 3 Surgery Department, Breast Unit, ICS Maugeri S.p.A. SB, via S. Maugeri 10, 27100 Pavia, Italy; 4 Nanomedicine laboratory, ICS Maugeri S.p.A. SB, via S. Maugeri 10, 27100 Pavia, Italy

## Abstract

Targeted therapies have profoundly changed the clinical prospect in human epidermal growth factor receptor 2 (HER2)-positive breast cancer. In particular, the anti-HER2 monoclonal antibody trastuzumab represents the gold standard for the treatment of HER2+ breast cancer patients. Its contribution in dampening cancer progression is mainly attributed to the antibody-dependent cell-mediated cytotoxicity (ADCC) rather than HER2 blockade. Here, multiple half chains of trastuzumab were conjugated onto magnetic iron oxide nanoparticles (MNP-HC) to develop target-specific and biologically active nanosystems to enhance anti-HER2 therapeutic potential. HER2 targeting was assessed in different human breast cancer cell lines, where nanoparticles triggered site-specific phosphorylation in the catalytic domain of the receptor and cellular uptake by endocytosis. MNP-HC induced remarkable antiproliferative effect in HER2+ breast cancer cells, exhibiting enhanced activity compared to free drug. Accordingly, nanoparticles induced p27kip1 expression and cell cycle arrest in G1 phase, without loosing capability to prime ADCC. Finally, MNP-HC affected viability of trastuzumab-resistant cells, suggesting interference with the resistance machinery. Our findings indicate that multiple arrangement of trastuzumab half chain on the nanoparticle surface enhances anticancer efficacy in HER2+ breast cancer cells. Powerful inhibition of HER2 signaling could promote responsiveness of resistant cells, thus suggesting ways for drug sensitization.

## Introduction

The overexpression or gene amplification of human epidermal growth factor receptor 2 (HER2) characterizes 20–30% of all breast cancers, which are classified as the HER2-positive subtype^1^. In this breast cancer population, the overexpression of HER2 triggers multiple downstream pathways required for the abnormal proliferation of cancer cells^2^. Being the disease addicted to HER2 for growth and proliferation, continuous inhibition of HER2 receptor represents the recommended treatment in case of HER2+ breast cancer^3^. The approval by the Food and Drug Administration of the first anti-HER2 antibody trastuzumab (TZ) has revolutionized the clinical scenario in HER2+ breast cancer leading to significantly improved disease-free and overall survival^4,5^. Since then, anti-HER2 strategies are used to control the disease and nowadays they include a number of targeted drugs, such as lapatinib, pertuzumab and trastuzumab emtansine^6,7^. Blockade of HER2 signaling is one of the key elements for improving the clinical outcome in HER2+ breast cancers, and several trials have investigated the efficacy of various combination of HER2-targeted drugs in addition to conventional chemotherapies^6^. Despite great progress in the field, the wide variability in response to therapy and the frequent onset of drug resistance in patients upon treatment still hamper the therapeutic success^8^. Furthermore, the need for long-lasting and optimal HER2 inhibition strongly encourages the development of new drugs and new techniques, particularly in case of resistant cells and in the metastatic disease.

Antibody-conjugated nanoparticles may combine specific recognition of tumor cells with the capability to act as delivery systems for active drugs^9^. Several bioconjugation strategies have been explored in order to achieve stable and oriented immobilization of targeting moieties, for optimizing detection of specific tumor biomarkers and obtaining targeted action^10,11^. In 2013, we analyzed the tumor targeting efficiency of multifunctional nanoconstructs bearing variants of TZ in a murine model of primary breast cancer^12^. We found that functionalization of small colloidal magnetic nanoparticles with the half chain of TZ (MNP-HC) provided increased stability and afforded long-term accumulation in the tumor, as compared to equal nanoparticles conjugated with the entire antibody or single-chain variable fragment (scFv) ligands. However, no functional studies have been performed so far for supporting the therapeutic performance associated with the observed tumor homing and improved retention mediated by the MNP-HC. Here, target specificity and biological activity of TZ-derived half chains immobilized on multivalent colloidal nanoparticles were investigated on breast cancer cell lines. Direct comparison with free TZ was made in order to characterize the efficacy of nanoparticles with respect to the same dosage of drug, following the idea that the spatial arrangement of the targeting moieties could be the key for antibody-ligand interaction and subsequent activity modulation. In addition, as the conjugation with colloidal nanoparticles seems to affect the therapeutic efficacy of TZ^13^, we explored the anticancer activity of MNP-HC both in HER2+ TZ-sensitive and resistant breast cancer cells.

## Results

### HER2 targeting by MNP-HC nanoparticles

**Figure 1:**
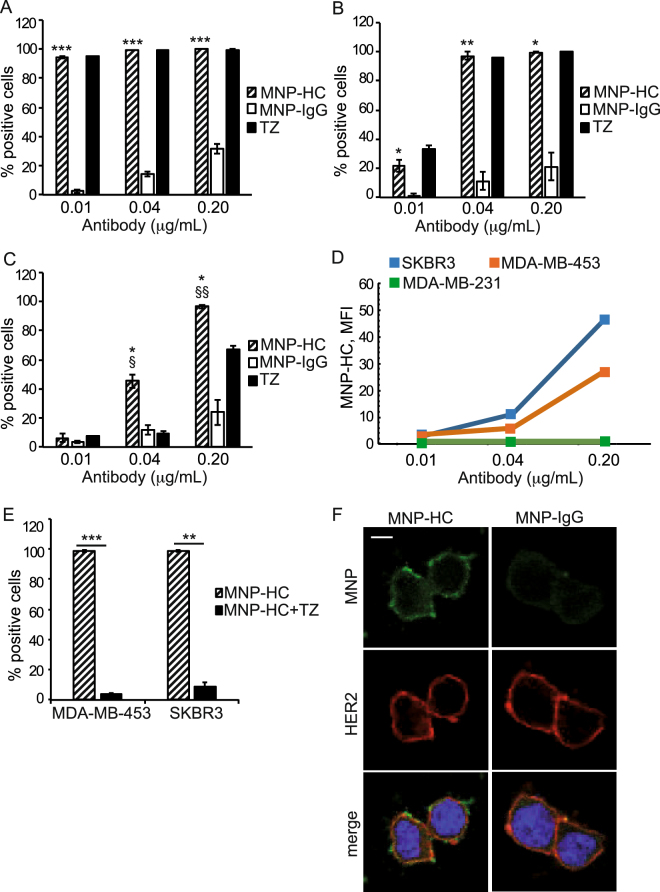
HER2 targeting by MNP-HC. MNP-HC binding to SKBR3 (**A**), MDA-MB-453 (**B**) and MDA-MB-231 (**C**) after 1 h incubation at 37 °C (n = 3). Asterisks indicate significance *vs*. MNP-IgG; § *vs*. TZ. (**D**) Mean fluorescence intensity of MNP-HC bound to the cells. (**E**) Competition assay in MDA-MB-453 and SKBR3 incubated with MNP-HC with or without an excess of TZ as competitor (n = 3). (**F**) Confocal laser-scanning micrographs of SKBR3 incubated for 30 min with MNP-HC or MNP-IgG (green) and stained for HER2 (red). Nuclei are stained with DAPI (blue). Scale bar: 10 μm.

MNP-HC were assessed for their capability to interact with multiple human breast cancer cell lines, classified as distinct carcinoma subtypes with different levels of HER2 expression (Table 1)^14^. The binding assay, performed at 37 °C, demonstrated a dose-dependent and target-related biorecognition of the cells (Fig. 1A–C). MNP-HC exhibited ≥ 97% binding to all the tested cell lines when incubated at a dose equal to 0.2 μg mL^−1^ of trastuzumab, while decreasing the dosage different outcomes were observed depending on the cell type. A complete binding was still detected in HER2-overexpressing SKBR3 cells (99.6% when using 0.04 μg mL^−1^ and 94.7% when using 0.01 μg mL^−1^), and in MDA-MB-453 at 0.04 μg mL^−1^ (97.3%). By contrast, reduced percentage of binding was recorded in the HER2-basal expressing MDA-MB-231 cells (45.3% at 0.04 μg mL^−1^, 6.4% at 0.01 μg mL^−1^) and in MDA-MB-453 at 0.01 μg mL^−1^ (21.7%), as following reduced expression of the target on the membrane of these cells. No detectable binding of MNP-HC was observed on HER2-negative MDA-MB-468 cells, unless using much higher doses (Supplementary Table 1). Binding specificity was attributed to the antibody-derived half chains coupled to the nanoparticle surface, as demonstrated by comparison with IgG-conjugated nanoparticles. Moreover, higher mean fluorescence intensities were observed in HER2-overexpressing cells, where dose-dependent mean fluorescence indicated increasing number of MNP-HC per single cell when concentrations rose (Fig. 1D). By contrast, in MDA-MB-231, the mean fluorescence intensity remained low and unvaried as concentrations increased, meaning reduced binding extent of MNP-HC when HER2 receptor was poorly available. Fluorescence intensity was low and constant even in case of IgG-conjugated nanoparticles, since their binding did not correlate with specific target (Supplementary Fig. 1). Binding profiles at 4 °C confirmed efficient HER2 targeting by MNP-HC (Supplementary Fig. 2). Competition assay was performed on SKBR3 and MDA-MB-453 cells by using free TZ as direct competitor for the binding to HER2 receptor. Obtained results demonstrated specific interaction of MNP-HC with HER2 receptor, as displayed by the ≥ 91% reduction in cell binding when an excess of unlabeled free TZ was added as a competitor (Fig. 1E). Targeting of HER2 receptor by MNP-HC was further supported by confocal microscopy on HER2-overexpressing cells incubated for 30 min with the nanoparticles (Fig. 1F). Fluorescent signal from MNP-HC was recovered attached to the cell membrane, where it overlapped with HER2 staining.

**Table 1:** Cell lines classification based on carcinoma subtype, PI3K and HER2 status.

Cell line	Tumor subtype	*ERBB2*	*PIK3CA*	HER2 expression
SKBR3	Luminal	Amplification	Wild type	3+
MDA-MB-453	Luminal	Amplification	Mutant	2+
MDA-MB-231	Basal-like	No amplification	Wild type	0–1+
MDA-MB-468	Basal-like	No amplification	Wild type	0
BT474TR	Luminal	Amplification	Mutant	3+
JIMT-1	Basoluminal	Amplification	Wild type	2+

### Site-specific HER2 phosphorylation upon nanoparticles exposure

**Figure 2:**
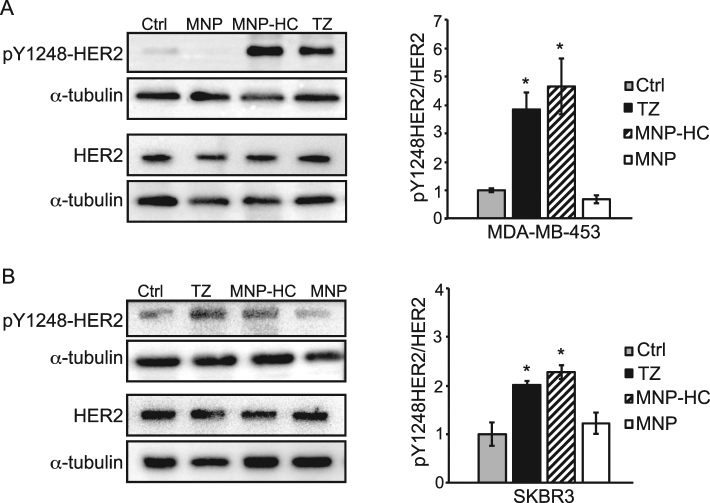
HER2 site-specific phosphorylation. Western blot analysis of pY1248-HER2 and total HER2 in MDA-MB-453 (**A**) and SKBR3 (**B**) treated with MNP-HC, TZ or unconjugated nanoparticles (MNP). Histograms show the densitometric quantification of pY1248-HER2 levels relative to total HER2, adjusted toward α-tubulin (n = 3). Asterisks indicate significance *vs*. untreated cultures (Ctrl).

In order to get insights into the targeted activity of MNP-HC on HER2 receptor, we assessed the capability of nanoparticles to induce phosphorylation of tyrosine residue 1248 (Y1248) within HER2 catalytic site. Indeed, it was demonstrated that specific binding of TZ is able to induce activation of HER2 tyrosine kinase by phosphorylation on Y1248 in sensitive breast cancer cells, and that this is associated to inhibition of cancer cell growth^15^. Therefore, site-specific HER2 phosphorylation could be considered a first read-out of TZ-mediated selective functionality. By treating HER2+ breast cancer cells with MNP-HC, we observed an increase in the ratio between Y1248-phosphorylated and total HER2 protein in MDA-MB-453 (Fig. 2A) and SKBR3 cells (Fig. 2B). No effect was induced by unconjugated nanoparticles, ascribing this effect to the TZ-derived moiety and not to other components of the nanoparticle core. HER2 phosphorylation levels upon MNP-HC incubation were comparable, or even higher, to those obtained in response to free TZ, thus indicating that the covalent conjugation to the nanoparticle did not affect the functionality of TZ drug.

### The intracellular trafficking of MNP-HC

**Figure 3:**
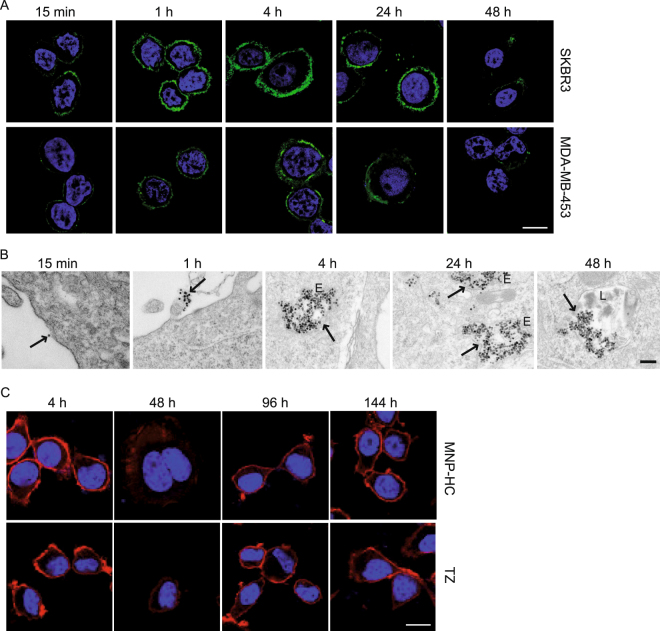
Nanoparticles intracellular trafficking. (**A**) Time course confocal microscopy of SKBR3 and MDA-MB-453 cells incubated with MNP-HC (green). Nuclei are stained with DAPI (blue). Scale bar: 10 μm. (**B**) TEM images of MNP-HC, pointed by arrows, in SKBR3 cells. E, endosomes; L, lysosomes. Scale bar: 200 nm. (**C**) Confocal laser-scanning micrographs of SKBR3 cells incubated with MNP-HC or free TZ, and stained for HER2 (red). Nuclei are stained with DAPI (blue). Scale bar: 10 μm.

Next, we investigated the cellular uptake of MNP-HC (Fig. 3A). Fluorescence corresponding to TZ half chain indicated a progressive accumulation of the nanoparticles at the cell membrane during the first hour of incubation, with faster kinetics in those cells with higher HER2 expression (SKBR3 > MDA-MB-453). After 4 and 24 h, some signal was detected inside the cell cytoplasm, in proximity to the plasma membrane, indicating nanoparticles internalization. At 48 h, the fluorescence decreased, indicating reduced rate of *de novo* interaction with the cell membrane, and probably degradation of the organic components of the nanoparticle, as previously reported^16^. Because of low HER2 expression, MDA-MB-231 showed only weak membrane signal, according to the mean fluorescence data observed in binding experiment (Supplementary Fig. 3). In parallel, the intracellular trafficking of the iron oxide core was followed by transmission electron microscopy (TEM) in SKBR3 cells (Fig. 3B). Dispersed nanocrystals were first observed outside the cells, and progressively attached to the cell membrane at 1 h, with specific enrichment on plasma membrane protrusions, according to previous observations of HER2 surface localization^17^. At 4 and 24 h, nanoparticles were compartimentalized in endosomal vesicles, mainly in the peri-membrane region, and later in lysosomes, thus suggesting the endocytic pathway as fate for the captured MNP-HC. Together with nanoparticles internalization, we observed a time-dependent downregulation of HER2 receptor on the plasma membrane of MNP-HC-treated, as well as TZ-treated cells (Fig. 3C). This effect was recovered in 48 h following treatment cessation.

### Enhanced antitumor activity in HER2+ breast cancer cells

**Figure 4:**
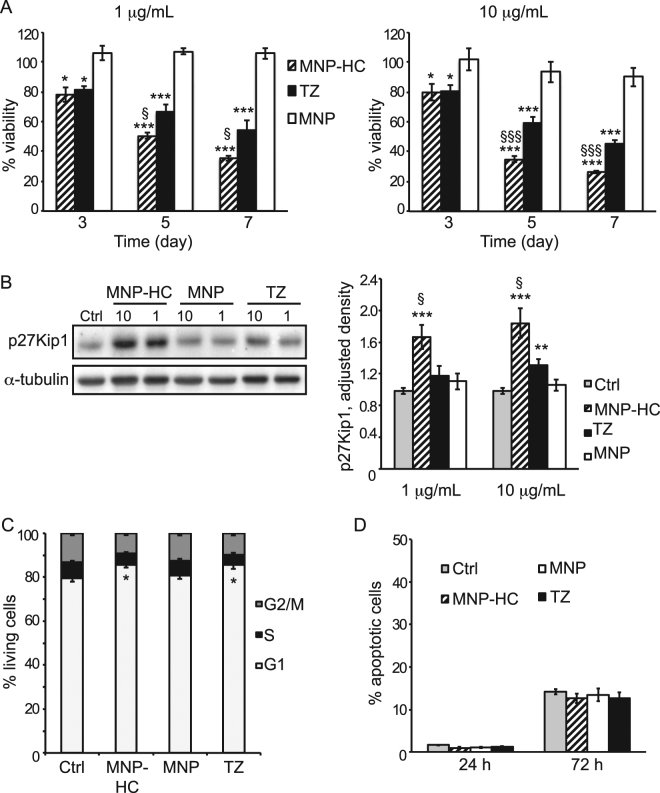
Antitumor activity in HER2+ cells. (**A**) Viability of SKBR3 treated for 3, 5 or 7 days with 1 (left panel) or 10 (right panel) μg mL^–1^ of free or nanoformulated TZ, or with equal concentrations of MNP (n = 6). Asterisks indicate significance *vs*. untreated cultures; § MNP-HC *vs*. TZ. (**B**) Western blot analysis of p27Kip1 expression in cells treated with 1 or 10 μg mL^–1^ of free or nanoformulated TZ, or with equal concentrations of MNP. Densitometric quantification of p27Kip1 was relative to α-tubulin (n = 4). Asterisks indicate significance *vs*. untreated cells (Ctrl); § MNP-HC *vs*. TZ. (**C**) Cell cycle analysis of SKBR3 treated with MNP-HC, MNP or TZ for 72 h. Results are mean percentage of cells in G1, S or G2/M phase (n = 7). Asterisks indicate significance *vs*. untreated cells (Ctrl). (**D**) The percentage of apoptotic cells was determined upon 24 or 72 h of treatment with MNP-HC, MNP or TZ (15 μg mL^−1^), and compared to untreated cells (Ctrl, n = 6).

Upon binding to the extracellular domain of HER2, TZ can potently suppress cancer cells growth, proliferation and survival by blocking HER2 signaling cascade and inducing cell cycle arrest^18^. In order to investigate whether MNP-HC maintain capability to exert this activity, we analyzed viability of HER2+ breast cancer cells upon treatment with MNP-HC or free TZ over one week. Results revealed a dramatic time-dependent reduction in the percentage of viable cells upon MNP-HC treatment: only 35.6% and 26.6% of cells were recovered alive after incubation with 1 or 10 μg mL^−1^ of nanoformulated TZ, respectively (Fig. 4A). MNP-HC improved the direct antitumor activity of free TZ by 34.4% when using 1 μg mL^−1^ and by 41.8% when using 10 μg mL^−1^. To exclude a toxic contribution due to the nanocrystal core, cells were also treated with equal amounts of unconjugated nanoparticles. The obtained results demonstrated a safe profile of the nanoparticle itself, thus attributing the efficacy of MNP-HC to specific HER2-targeted activity.

We also assessed the effect of the nanoformulation on one of the key effectors of HER2-targeted therapies, the cyclin-dependent kinase inhibitor p27Kip1^19^. Western blotting performed on total cell extracts after 24 h treatment with free TZ or MNP-HC revealed a remarkable increase in p27Kip1 protein upon MNP-HC incubation, with statistical significance *versus* free TZ (Fig. 4B). The nanoconjugation achieved efficient induction of p27Kip1 expression even at 1 μg mL^−1^, a dose resulting ineffective when using TZ alone. No effect was detected upon treatment with unconjugated nanoparticles, supporting the involvement of TZ half chain in the HER2-targeted activity. According to the increase in p27Kip1 expression, cell cycle arrest in G1 phase occurred in response to MNP-HC (Fig. 4C). This effect was comparable to that achieved with an equal dose of free TZ, while unconjugated nanoparticles did not affect the physiological cell cycle. Reduced proliferation and cell cycle arrest were not associated with increased apoptosis of breast cancer cells (Fig. 4D), supporting the global biosafety of MNP nanoparticles.

Taken together, these results showed that MNP-HC block HER2-mediated intracellular signaling and interfere with HER2-driven cancer cell proliferation, thus triggering a direct and potent antitumor efficacy in HER2+ breast cancer cells.

### The ADCC mechanism of action

Besides direct inhibition of HER2-mediated signaling, TZ promotes a specific mechanism termed “antibody-dependent cell-mediated cytotoxicity” (ADCC) through the activation of natural killer (NK) cells^20,21^. The recognition of TZ Fc-γ bound to HER2 by CD16 receptor expressed onto NK cells induces a signal cascade leading to the release of lytic granules against cancer cells^22^.

**Figure 5:**
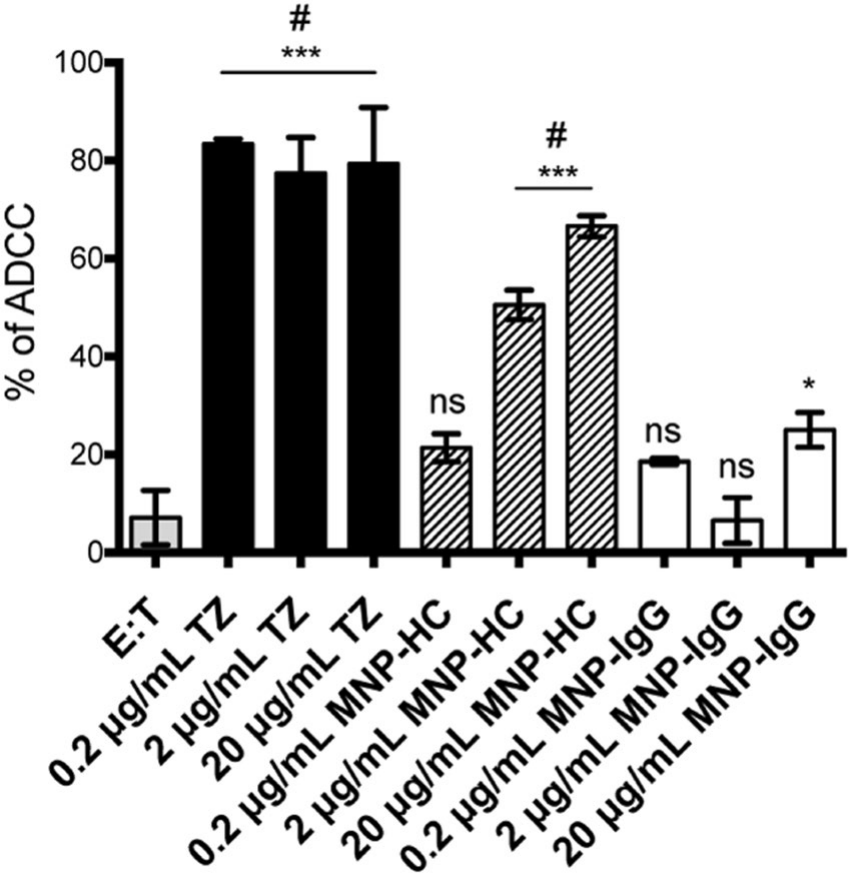
ADCC mechanism induced by TZ, MNP-HC and MNP-IgG on SKBR3. Data are percentage of mean ± SD of three independent replicates. *p < 0.05 vs E:T; ***p < 0.001 *vs*. E:T; ^#^p < 0.001 *vs*. MNP-IgG; ns *vs*. E:T.

To evaluate whether MNP-HC were still able to activate ADCC mechanism even if TZ was presented as a half chain fragment, we treated SKBR3 cells with MNP-HC at different concentrations (0.2, 2 and 20 μg mL^−1^) and compared effect with free TZ and nonspecific IgG-conjugated nanoparticles used as positive and negative controls, respectively. Upon addition of peripheral blood mononuclear cell (PBMC) population, which contained approximately 5% NK cells, lysis of tumor cells induced by NK cells was quantified using a lactate dehydrogenase (LDH) assay, which correlates the release of LDH enzyme by tumor cells to cytolytic activity of NK cells. Figure 5 confirms the strong effectiveness of TZ to induce ADCC even at the lowest concentration tested (0.2 μg mL^−1^). MNP-HC showed a dose-dependent activity in the induction of ADCC. This result was worthy of note, considering that to our knowledge this experiment represents the first example in which a fragment only of a monoclonal antibody immobilized onto the surface of colloidal nanoparticles was able to elicit ADCC activation, suggesting that nanoconjugate-mediated ADCC does not necessitate the presence of the whole antibody. As expected, cytolysis was not observed when MNP-IgG were used in place of MNP-HC.

### Impact of MNP-HC on trastuzumab-resistant cells

**Figure 6:**
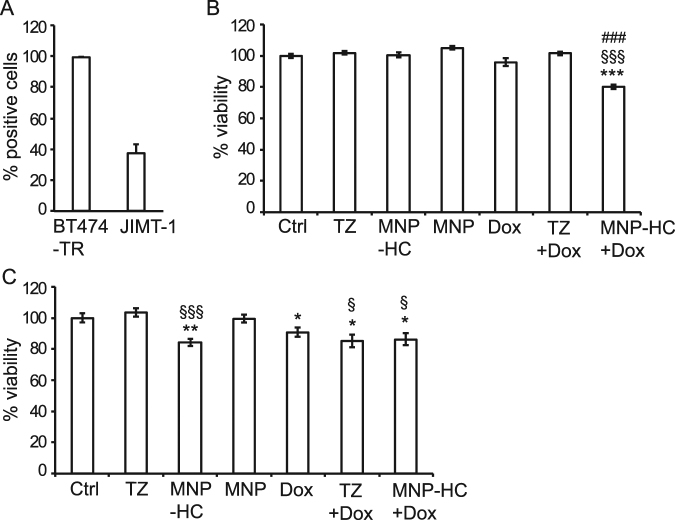
Activity in TZ-resistant cells. (**A**) MNP-HC binding to BT474TR and JIMT-1 after 1 h incubation with 0.2 μg mL^–1^ of nanoformulated TZ at 37 °C (n = 3). (**B**,**C**) Viability of JIMT-1 (**B**) or BT474TR (**C**) treated with sequential combination of free or nanoformulated TZ (10 μg mL^–1^) and doxorubicin (Dox, 0.1 μM), or with single agents. Asterisks indicate significance *vs*. Ctrl; § *vs*. TZ; # *vs*. Dox (n = 6).

To explore to what extent MNP-HC could be effective therapeutic agents, we analyzed their capability to interfere with drug resistance, as a stand-alone treatment or in combination with chemotherapy. To this purpose, we used two breast cancer cell lines both characterized by HER2 overexpression and by TZ resistance: BT474TR and JIMT-1 (Table 1). We first checked the capability of MNP-HC to interact with these cells (Fig. 6A), finding high percentage of binding in BT474TR (99.7%), and a lower, although not negligible, binding in JIMT-1 (37.1%). Such a difference could sound disappointing considering the high HER2 expression level in both cell types; however, our data agree with previous publications reporting reduced binding of TZ to JIMT-1 cells, which was attributed to epitope masking^23^. Next, we analyzed the antitumor potential of a sequential combination of MNP-HC and doxorubicin, a clinically-used anthracycline and excellent pilot chemotherapeutic drug. Measurement of cell viability revealed that the combined treatment induced a significant antitumor activity in JIMT-1 cells (p < 0.001), whereas none of the antitumor agents resulted effective when administrated alone (Fig. 6B). As expected, sustained viability of JIMT-1 cells was maintained upon treatment with TZ with or without doxorubicin, indicating failure for TZ alone to both affect viability of resistant cells and induce chemosensitivity. In BT474TR, inefficacy was confirmed for treatment with TZ; however, treatment with equal dose of MNP-HC was able to slightly but significantly affect cell viability, demonstrating an average 16% reduction after 3 days of treatment (p < 0.001 *vs*. TZ) (Fig. 6B). In this case, the sequential treatment with doxorubicin did not further enhance the antitumor potential of MNP-HC, demonstrating no synergistic effect. In both JIMT-1 and BT474TR cell lines the inhibition of cell growth was not associated with increased phosphorylation of Y1248-HER2 (Supplementary Fig. 4), thus suggesting that diverse molecular mechanisms should be involved in driving the different response to MNP-HC in case of TZ resistance.

## Discussion

Trastuzumab has changed the natural history of HER2+ breast cancer, leading to a strong improvement in overall survival^6,24^. The antiproliferative mechanism of TZ has not been completely elucidated, and *in vivo* a prominent role could be hold by ADCC rather than a specific impact on HER2-mediated intracellular signaling^25^. Indeed, after binding to HER2, the Fc portion of TZ antibody stimulates the antitumor immunity and the cytotoxic activity of natural killer cells, which recognize TZ-coated breast cancer cells. However, TZ may also exert a direct antitumor activity by blocking HER2-mediated signaling. In the tumor cells, TZ binding on the extracellular domain of HER2 inhibits relevant growth and proliferation pathways, such as those related to PI3K/Akt and MAPK, thus reducing cancer cell division and tumor progression^26^. Strengthening HER2 targeting and optimizing the activity on downstream signaling could achieve a more potent anticancer activity and improve the global therapeutic efficacy of TZ. In this study, we show that multiple and oriented immobilization of TZ-derived ligands on the surface of colloidal nanoparticles improves the antitumor performance on HER2+ breast cancer cells by acting predominantly through blockade of cell signaling.

MNP-HC nanoparticles showed efficient targeting of HER2, with induction of site-specific phosphorylation in the catalytic domain of the receptor and rapid cellular uptake by endocytosis. The multivalent exposure of TZ half chains also led to enhanced antitumor efficacy against HER2+ breast cancer cells, dramatically decreasing cancer cell viability. This effect was associated with marked induction of p27Kip1 expression and cell cycle arrest in G1 phase in TZ-sensitive cells, thus indicating that once immobilized on the nanoparticle surface, the antibody-derived ligands maintain their specificity for HER2 and block HER2-driven cancer cell proliferation. Enhanced activity of MNP-HC *vs.* TZ could find explanation on the multiple immobilization of antibody derivatives on carriers with narrow size, which could be suitable for targeting of receptor clustering. Amplified recognition and selectivity was likely triggered by ligands multimerization and spatial arrangement on the surface of nanoparticles, upon orientation-controlled conjugation strategy^27,28^. Indeed, treatment of HER2+ cells with MNP-HC resulted in sustained phosphorylation of tyrosine residue 1248 even at low dosage of therapeutic drug, as compared to free TZ. This phosphorylation has been shown to provide recognition sites for intracellular signaling intermediates, which link TZ-bound HER2 to inhibition of downstream transduction cascades that ultimately result in gene expression changes and inhibition of cancer cell proliferation^15^. By boosting HER2 recognition and impact on HER2 receptor, MNP-HC induced reduction in cancer cell viability and arrest of the cell cycle, thus enhancing anti-HER2 efficacy of TZ antibody.

Our *in vitro* results also indicated that MNP-HC did not loose ADCC property, which normally covers the majority of TZ activity against cancer cells. In this case, efficiency was lower as compared to TZ alone, likely due to the half Fc portion presented by MNP-HC. Despite improvement of antitumor performance by MNP-HC was related to a greater impact on intracellular signaling transduction, maintenance of ADCC capability suggests additional potential for the nanocomplex in stimulating the host antitumor immunity.

A significant proportion of HER2+ breast cancer patients is affected by primary TZ-resistance, and up to 70% of patients who previously responded to TZ develop resistance to treatment in the first year^29^. Among multiple mechanisms of resistance to TZ, some rely on proteolysis or masking of HER2, hindering TZ binding^30^. Furthermore, highly glycosylated membrane proteins such as Mucin-4 could mask HER2 binding sites for TZ^31^. We reasoned that it could have been possible that optimizing interaction with cancer cells by spatially-oriented immobilization on nanocarriers might weaken drug resistance. In this study, we found that powerful inhibition of HER2 signaling by TZ-conjugated nanoparticles could favor responsiveness of drug resistant cells. In particular, treatment with MNP-HC achieved a significant reduction of cell viability in BT474TR TZ-resistant cell line. In addition, in JIMT-1 cells, a resistant cell line described to be highly refractory to TZ treatment, we observed that MNP-HC were able to sensitize cells to chemotherapy. Indeed, therapeutic resistance in breast cancer includes multiple molecular mechanisms involved in the onset of resistance and the activation of alternative cell type-dependent pathways. Main concern could be represented by the status of *PIK3CA*, which could accelerate tumor progression, alter intrinsic phenotype of HER2+ cancers, and cause resistance to anti-HER2 therapies^32^. Therefore, by suggesting multifaceted properties of MNP-HC efficacy on different TZ-resistant breast cancer cell lines, our findings encourage further studies on the mechanisms involved in preventing or bypassing resistance.

Several studies have previously demonstrated the great potential of active functionalization of nanoparticles toward cancer cells, and TZ-mediated targeting has been broadly investigated. However, functional implications of such targeting remain almost unexplored and are still mostly unclear. In the present study, we have demonstrated that immobilization of TZ half chain on colloidal nanoparticles not only provided an excellent homing and internalization in HER2+ breast cancer cells, but also preserved a direct anti-HER2 activity by inhibiting HER2-related intracellular signaling. Here a bare magnetic nanoparticle was used to avoid any bias in the analysis of the anticancer efficacy of the nanoconjugate. However, such targeted nanoparticles could be loaded with cytotoxic drugs, providing a novel strategy based on a multi-acting HER2-targeted nanodrug against breast cancer. In-one compound multiple action could contribute to overcome major limitations of currently used anti-HER2 treatments, such as chemoresistance and the need for therapeutic switch to different options.

## Methods

### Nanoparticles production

MNP-HC were synthesized following the protocol previously set up in our laboratory^12^. Briefly, TZ dissolved in EDTA–PBS (1 mg mL^−1^) was added to the 2-mercaptoethanolamine kit (MEA, Thermo Fisher Scientific) to reduce the disulfide bridges between the two heavy chains of the IgG. The obtained half-chain antibody portions (HCs) were immediately added to MNP (1 mg) and incubated at room temperature for 1 h. The remaining PDP functional groups were saturated with excess PEG500-SH. Excess reagents were removed by dialysis, and MNP-HC were collected. Nonspecific rabbit IgG-MNP, used as control, were prepared according to the protocol above.

### Cell culture

SKBR3 and MDA-MB-231 cell lines were purchased by ATCC-LGC Standards and Caliper LifeSciences, respectively. MDA-MB-453 were a generous gift from Dr. E. Tagliabue (IRCCS Istituto Nazionale dei Tumori, Milano, Italy), while JIMT-1 and BT474TR were kindly provided by Dr. L. Santarpia (IRCCS Humanitas Clinical and Research Center, Milano, Italy). SKBR3 were cultured in 50% high glucose Dulbecco’s Modified Eagle Medium (DMEM), 50% Ham’s F12 Nutrient Mixture; MDA-MB-453, JIMT-1 and BT474TR in high glucose DMEM; MDA-MB 231 in Minimum Essential Medium. All media were supplemented with 10% heat inactivated fetal bovine serum (FBS), 2 mM l-glutamine, 100 U mL^−1^ penicillin, 0.1 mg mL^−1^ streptomycin (Euroclone). All cell lines grew at 37 °C in a humidified atmosphere containing 5% CO_2_ and were subcultured prior to confluence using trypsin/EDTA.

### Cell binding assay

For in-plate cell binding assay, 3 × 10^5^ cells were seeded on a 6-well plate and incubated for 1 h at 37 °C with 0.01, 0.04, 0.2 µg mL^−1^ of free or nanoformulated TZ, or with corresponding concentrations of MNP-IgG dissolved in culture medium. Cells were washed three times with phosphate buffer saline (PBS), incubated for 15 min in 2% bovine serum albumin (BSA, Sigma-Aldrich), 2% goat serum (Euroclone) in PBS and stained with appropriate secondary antibody conjugated with Alexa Fluor 488 (0.5 μg/sample, Thermo Fisher Scientific) in blocking buffer for 15 min at room temperature. After PBS washing, cells were analyzed by CytoFLEX flow cytometer (Beckman Coulter), by gating on viable cells and acquiring 10,000 events for each analysis. The level of binding *per* single cellular event was determined by geometric mean fluorescence intensity. Cells incubated with the secondary antibody only were used to set the positivity region. For in-tube cell binding assay, 5 × 10^5^ cells were collected in plastic tubes and incubated for 2 h at 4 °C in 0.3% BSA-PBS supplemented with 0.01, 0.04, 0.2 µg mL^−1^ of free or nanoformulated TZ, or with corresponding concentrations of MNP-IgG. Cells were washed three times with PBS, and processed as described above.

### Competition assay

Cells (5 × 10^5^) were incubated for 30 min at 37 °C in 0.3% BSA-PBS supplemented with 0.1 µg mL^−1^ of nanoformulated TZ previously labeled with fluorescein isothiocyanate (FITC) in presence or absence of 100-fold molar excess of free unlabeled TZ. Cells were washed three times with PBS and analyzed by CytoFLEX. After gating on viable and single cells, 10,000 events were acquired for each analysis. Untreated cells were used to set the positivity region.

### Protein analysis

For the analysis of Y1248-HER2 phosphorylation, 3 × 10^5^ cells were serum-starved in medium containing 0.1% FBS overnight at 37 °C, and incubated for 1 h at 37 °C with 4 µg mL^−1^ of nanoformulated TZ or corresponding concentration of unconjugated nanoparticles. For the analysis of p27Kip1, cells were incubated for 24 h at 37 °C with 1 or 10 µg mL^−1^ of nanoformulated TZ or corresponding concentrations of unconjugated nanoparticles in complete medium. Untreated cells were used as reference for basal protein status, while cells treated with free TZ were used as positive control. After the incubations, cells were washed with PBS, lysed in Triton lysis buffer (20 mM Tris-HCl pH 7.6, 150 mM NaCl, 1 mM EDTA, 10% Glycerol, 1% Triton X-100), containing 4% Protease Inhibitor Cocktail (Roche), 1 mM PMSF (Sigma-Aldrich), 1 mM Na_3_VO_4_ (Sigma-Aldrich), 10 mM NaF (Sigma-Aldrich), and cleared at 17,100 × g for 15 min at 4 °C. Protein content was quantified by Bradford method.

### Immunofluorescence and confocal laser scanning microscopy

Cells (0.5 × 10^5^) were seeded on glass coverslips in a 24-well plate and incubated with 0.02 mg mL^−1^ of MNP-HC or MNP-IgG or corresponding concentrations of TZ for the indicated time points at 37 °C. When required, treatment was stopped after 48 h and cells were cultured for additional 96 h in fresh medium. At the indicated time points, cells were washed three times with PBS, fixed for 5 min with 4% paraformaldehyde (Sigma-Aldrich), permeabilized for 10 min with 0.1% Triton X-100, and incubated in 2% BSA, 2% goat serum in PBS for 2 h at room temperature. For analysis of cell uptake, MNP-HC were detected by Alexa Fluor 488-conjugated anti-human secondary antibody for 2 h at room temperature. For analysis of HER2 membrane expression, cells were immunodecorated overnight at 4 °C with anti-HER2/ErbB2 antibody (clone 29D8, Cell Signaling Technology, Inc.) diluted in blocking buffer, washed three times with PBS and incubated with Alexa Fluor 546-conjugated secondary antibody (Thermo Fisher Scientific) for 2 h at room temperature. Nuclei were stained with DAPI (0.1 μg mL^−1^). Coverslips were mounted in Prolong Gold antifade reagent (Thermo Fisher Scientific) and images were acquired with Leica SP8 microscope confocal system equipped with laser excitation lines 405 nm, 488 nm, 535 nm and 633 nm. Images were acquired with 63 × magnification oil immersion lens.

### Transmission electron microscopy

Cells (9 × 10^5^) were incubated with 0.02 mg mL^−1^ of MNP-HC for 15 min or 1, 4, 24, 48 h at 37 °C. Cells were washed three times with PBS, collected in 1.5 mL eppendorf, fixed in 2.5% glutaraldehyde (Electron Microscopy Sciences) in 0.1 M phosphate buffer, pH 7.2, for 2 h, rinsed with phosphate buffer, post-fixed in 1.5% osmium tetroxide (Electron Microscopy Sciences) for 2 h, dehydrated by 70, 90 and 100% EtOH, and embedded in epoxy resin (PolyBed 812 Polysciences Inc). Ultrathin sections were examined by TEM (Zeiss EM109).

### Western blotting

A 20 μg protein aliquot was resuspended in Laemmli buffer, resolved on polyacrylamide gels under reducing conditions and transferred to polyvinylidine difluoride membranes (Immobilon-P, EMD Millipore Corporation). Membranes were blocked with 5% skim milk or 5% BSA in Tris buffer saline (TBS) with 0.1% Tween-20 (Sigma-Aldrich) for 1 h at room temperature, and incubated with appropriate primary antibodies: anti-phospho-HER2/ErbB2 Tyr1248 (Cell Signaling Technology, Inc.), anti-HER2/ErbB2 (clone 29D8, Cell Signaling Technology, Inc.), anti-p27 KIP1 (Abcam), or anti-α-tubulin (Sigma-Aldrich). Antibodies conjugated to horseradish peroxidase (Abcam) were used as secondary antibodies, and chemiluminescence reaction was developed with the ECL star kit (Euroclone). Densitometric analysis of protein bands was performed with ImageJ software.

### Cell cycle analysis

Cells (10^5^) were seeded on a 12-well plate and incubated for 72 h at 37 °C with 15 µg mL^−1^ of nanoformulated TZ or corresponding concentration of unconjugated nanoparticles. Cells were washed twice with PBS, fixed with cold ethanol 70% for 1 h at room temperature, and labeled with 80 µg mL^−1^ Propidium Iodide (Sigma-Aldrich), 100 µg mL^−1^ RNase A (Sigma-Aldrich) and 0.1% Triton X-100 (Sigma-Aldrich) in PBS. After gating on viable and single cells, 20,000 events were acquired with CytoFLEX. Untreated cells and cells treated with free TZ were used as negative and positive control, respectively.

### Cell viability assay

Cells (3 × 10^3^) were seeded on a 96-well plate and incubated with 1 or 10 µg mL^−1^ of free or nanoformulated TZ, or with corresponding concentrations of unconjugated nanoparticles, by replacing incubation medium every two days. After 3, 5 and 7 days of incubation, cells were washed with PBS and tested with CellTiter 96^®^ AQ_ueous_ Non-Radioactive Cell Proliferation Assay (Promega Corporation), according to the manufacturer’s instructions. For the combination treatment with doxorubicin, 5 × 10^3^ cells were seeded on a 96-well plate and incubated for 24 h with 10 µg mL^−1^ of free or nanoformulated TZ, or with corresponding concentrations of unconjugated nanoparticles. Then, medium was replaced by 0.1 µM of doxorubicin hydrochloride (Pfizer) for additional 48 h. Absorbance was read using a testing wavelength of 490 nm and a reference wavelength of 630 nm. The results were normalized on viability of untreated samples (set at 100% viability).

### Cell death assay

Cells (10^5^) were treated for 24 or 72 h with 15 µg mL^−1^ of free or nanoformulated TZ, or with corresponding concentration of unconjugated nanoparticles, washed twice with PBS and treated with Annexin V-PE-Cy5 Apoptosis Detection Kit (BioVision) following the manufacturer’s protocol. Cells were analyzed within 15 min with CytoFLEX, by gating on viable cells and acquiring 20,000 events for each analysis.

### Antibody-dependent cell-mediated cytotoxicity

ADCC was evaluated using CytoTox 96® Non-Radio-active Cytotoxicity Assay (Promega Corporation), by adding effector (E-PBMCs) onto target cells (T-SKBR3, 5×10^3^) at a E:T ratio of 40:1 in 96-wells. Firstly, target cells were coated with 0.2, 2 and 20 μg mL^−1^ of free or nanoformulated TZ for 30 min at 4 °C in RPMI-1640 medium. As control, cells were coated with equal concentrations of MNP-IgG. Then, PBMCs were added and after 4 h at 37 °C, LDH release from target cells was measured by Ensight™ multimode plate reader (Perkin Elmer) setting absorbance wavelength at 490 nm. Percentage of ADCC was calculated as follows:

$$ \% \,{\boldsymbol{specific}}\,{\boldsymbol{lysis}}=\frac{{\boldsymbol{experimental}}\,{\boldsymbol{abs}}-{\boldsymbol{effector}}\,{\boldsymbol{spontaneous}}-{\boldsymbol{target}}\,{\boldsymbol{spontaneous}}}{{\boldsymbol{target}}\,{\boldsymbol{maximum}}-{\boldsymbol{target}}\,{\boldsymbol{spontaneous}}}\times \,{\bf{100}}$$

where *target maximum* is the absorbance value of target cells upon lysis; *target and effector spontaneous* is the absorbance value of target and effectors cells, respectively.

### Statistical analysis

Statistical analyses were conducted using two-tailed Student’s t-test. Unless otherwise specified, plots show mean ± standard error (SE) and the statistical significance is set as follows: *p < 0.05, **p < 0.01, ***p < 0.001.

### Ethical issue

Blood collection for ADCC studies was authorized by the Ethical Committee of the University of Milano-Bicocca as prot. 351 (protocol number 0078634/17) and was conducted in accordance with the International Conference on Harmonization (ICH) Good Clinical Practice (GCP) guidelines. All patients that decided to participate signed a written informed consent prior to inclusion in the study.

## Electronic supplementary material

Supplementary Information
