# Supplementary material for: Multivalent exposure of trastuzumab on iron oxide nanoparticles improves antitumor potential and reduces resistance in HER2-positive breast cancer cells
Source: Sci Rep. 2018 Apr 26;8:6563. doi: 10.1038/s41598-018-24968-x (PMC5920071; doi:10.1038/s41598-018-24968-x)

Multivalent exposure of trastuzumab on iron oxide nanoparticles improves antitumor potential and reduces resistance in HER2-positive breast cancer cells

Marta Truffi[1,+], Miriam Colombo[2,+], Luca Sorrentino[1], Laura Pandolfi[2], Serena Mazzucchelli[1], Francesco Pappalardo[2], Chiara Pacini[2], Raffaele Allevi[1], Arianna Bonizzi[1], Fabio Corsi[1,3,4*], Davide Prosperi[2,4*]

[1] Department of Biomedical and Clinical Sciences "L. Sacco", University of Milano, via G. B. Grassi 74, 20157 Milano, Italy
[2] NanoBioLab, Department of Biotechnologies and Biosciences, University of Milano-Bicocca, Piazza della Scienza 2, 20126 Milano, Italy
[3] Surgery Department, Breast Unit, ICS Maugeri S.p.A. SB, via S. Maugeri 10, 27100 Pavia, Italy
[4] Nanomedicine laboratory, ICS Maugeri S.p.A. SB, via S. Maugeri 10, 27100 Pavia, Italy.

[+] These Authors contributed equally to the study.

* Corresponding Authors:
Fabio Corsi
E-mail: fabio.corsi@unimi.it; Phone: +39.02.5031.9850; Fax: +39.02.5031.9846

Davide Prosperi
E-mail: davide.prosperi@unimib.it; Phone: +39.02.6448.3302

Supplementary Table 1: Flow cytometric analysis of MNP-HC binding to MDA-MB-468 at 37 °C in culture medium. Cells were incubated with 0.2, 1 and 4 μg mL^{-1} of nanoformulated TZ. Untreated cells incubated with secondary antibody only were used to set the positivity region. Data represent means ± SE (n = 3).

μg mL^{-1}	0	0.2	1	4
positive events (%)	0.37 ± 0.107	0.42 ± 0.098	0.34 ± 0.055	5.46 ± 0.600

Supplementary Fig. 1: Mean fluorescence intensity of MNP-IgG after binding to SKBR3, MDA-MB-453 and MDA-MB-231 at increasing concentrations (n = 3).

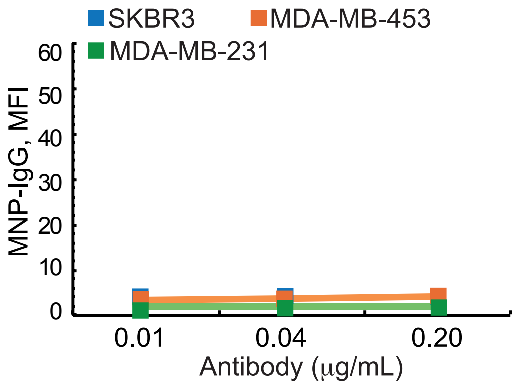

Supplementary Fig. 2: Flow cytometric analysis of MNP-HC binding to SKBR3 (a), MDA-MB-453 (b) and MDA-MB-231 (c) cells after 2 hours incubation at 4 °C in 0.3% BSA-PBS. Cells were incubated with 0.01, 0.04 or 0.2 µg mL^{-1} of free or nanoformulated TZ, or with corresponding concentrations of aspecific nanoparticles (MNP-IgG). Data represent means ± SE (n = 3). *p<0.05,**p<0.01,***p<0.001 *versus* MNP-IgG; , §§p<0.01, §§§p<0.001 *versus* free TZ.

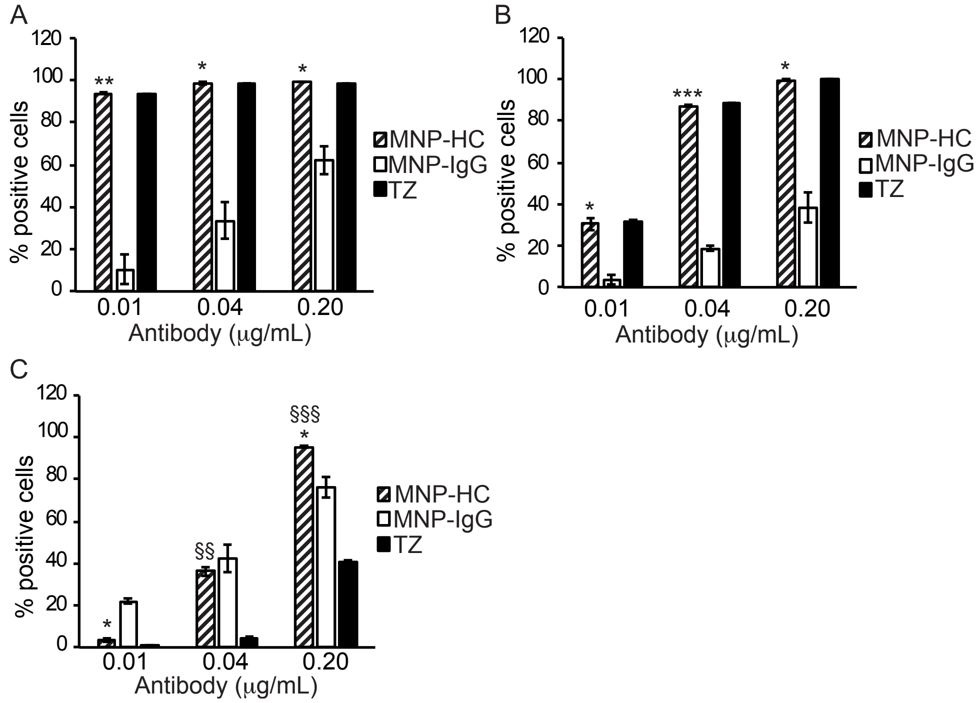

Supplementary Fig. 3: Time course confocal microscopy of MDA-MB-231 cells incubated with MNP-HC (green). Nuclei are stained with DAPI (blue). Scale bar: 10 μm.

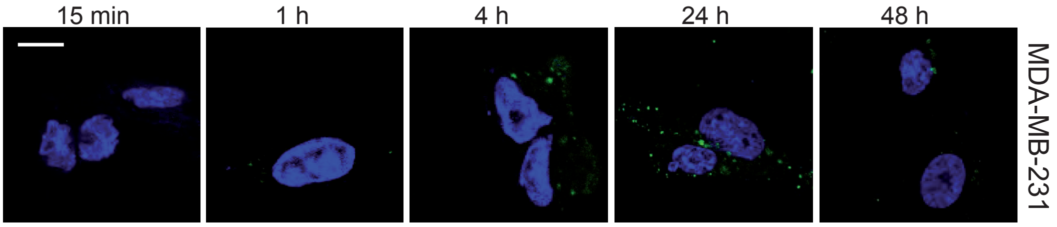

Supplementary Fig. 4: Western blot analysis of phosphorylated HER2 (pY1248-HER2) and total HER2 in whole protein extracts from JIMT-1 (a) and BT474TR (b) cells treated with 1 or 10 μg mL^{-1} of nanoformulated TZ (MNP-HC) or corresponding amount of unconjugated nanoparticles (MNP), as compared to untreated cells (Ctrl). Alpha-tubulin was used as loading control. Black lines separate blots cropped from different parts of the same gel.

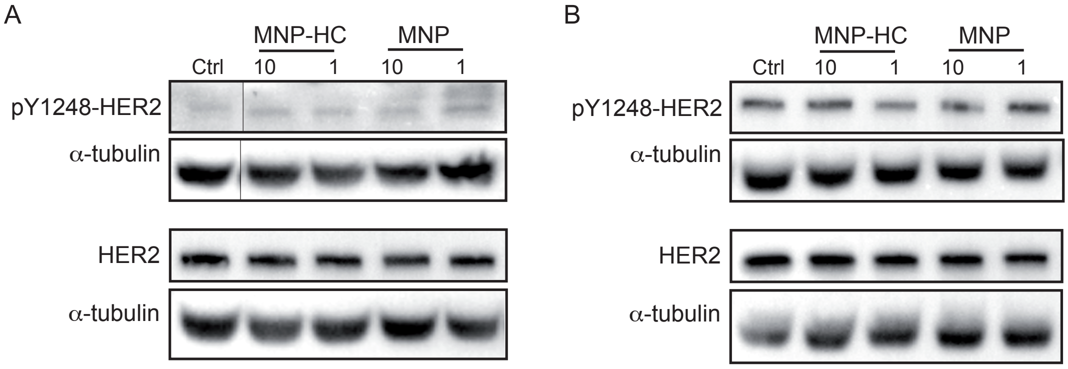

Supplementary Fig. 5: Uncropped blots for Figure 2A, B. Red marks indicate molecular weight of protein marker.

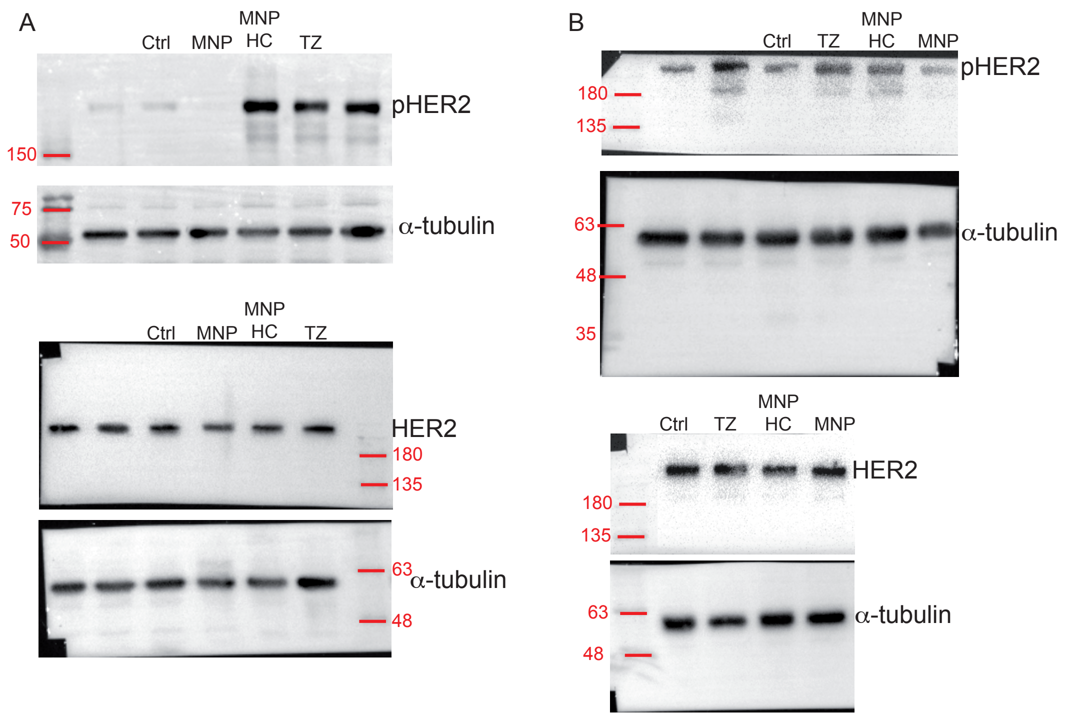

Supplementary Fig. 6: Uncropped blots for Figure 4B. Red marks indicate molecular weight of protein marker.

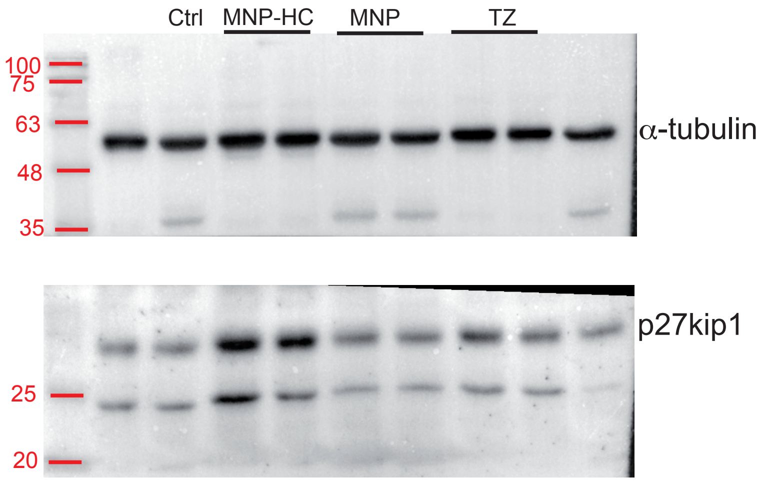

Supplementary Fig. 7: Uncropped blots for Supplementary Figure 2A, B. Red marks indicate molecular weight of protein marker.

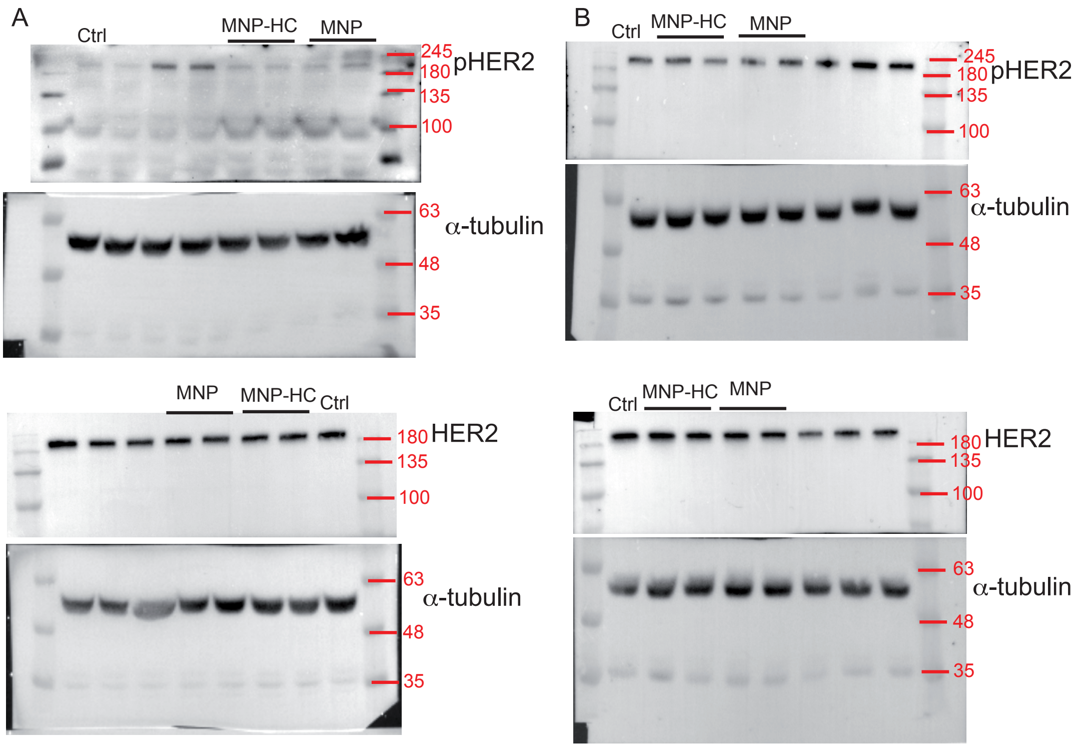

Supplement: Supplementary file 1 — Supplementary Information [file 41598_2018_24968_MOESM1_ESM.pdf]
